# Impaired Angiogenesis during Fracture Healing in GPCR Kinase 2 Interacting Protein-1 (GIT1) Knock Out Mice

**DOI:** 10.1371/journal.pone.0089127

**Published:** 2014-02-19

**Authors:** Guoyong Yin, Tzong-Jen Sheu, Prashanthi Menon, Jinjiang Pang, Hsin-Chiu Ho, Shanshan Shi, Chao Xie, Elaine Smolock, Chen Yan, Michael J. Zuscik, Bradford C. Berk

**Affiliations:** 1 Aab Cardiovascular Research Institute and the Department of Medicine, University of Rochester Medical Center, Rochester, New York, United States of America; 2 Center for Musculoskeletal Research and the Department of Orthopaedics and Rehabilitation, University of Rochester Medical Center, Rochester, New York, United States of America; 3 Orthopaedic Department, The First Affiliated Hospital of Nanjing Medical University, Jiangsu, China; INSERM U1059/LBTO, Université Jean Monnet, France

## Abstract

G protein coupled receptor kinase 2 (GRK2) interacting protein-1 (GIT1), is a scaffold protein that plays an important role in angiogenesis and osteoclast activity. We have previously demonstrated that GIT1 knockout (GIT1 KO) mice have impaired angiogenesis and dysregulated osteoclast podosome formation leading to a reduction in the bone resorbing ability of these cells. Since both angiogenesis and osteoclast-mediated bone remodeling are involved in the fracture healing process, we hypothesized that GIT1 participates in the normal progression of repair following bone injury. In the present study, comparison of fracture healing in wild type (WT) and GIT1 KO mice revealed altered healing in mice with loss of GIT1 function. Alcian blue staining of fracture callus indicated a persistence of cartilagenous matrix in day 21 callus samples from GIT1 KO mice which was temporally correlated with increased type 2 collagen immunostaining. GIT1 KO mice also showed a decrease in chondrocyte proliferation and apoptosis at days 7 and 14, as determined by PCNA and TUNEL staining. Vascular microcomputed tomography analysis of callus samples at days 7, 14 and 21 revealed decreased blood vessel volume, number, and connection density in GIT1 KO mice compared to WT controls. Correlating with this, VEGF-A, phospho-VEGFR2 and PECAM1 (CD31) were decreased in GIT1 KO mice, indicating reduced angiogenesis with loss of GIT1. Finally, calluses from GIT1 KO mice displayed a reduced number of tartrate resistant acid phosphatase-positive osteoclasts at days 14 and 21. Collectively, these results indicate that GIT1 is an important signaling participant in fracture healing, with gene ablation leading to reduced callus vascularity and reduced osteoclast number in the healing callus.

## Introduction

Fracture healing is a complex process involving an early inflammatory phase, recruitment, expansion and differentiation of mesenchymal cells, and production of cartilage and bone matrix in a temporally regulated manner [1]–[3]. After fracture, the repair process begins with hematoma formation and an inflammatory response [2]. In this early inflammatory phase, lack of blood vessels causes a regional hypoxic environment leading to the formation of a cartilagenous template that initiates a process of differentiation that recapitulates endochondral ossification [4]. Included are the proliferation and differentiation of mesenchymal progenitor cells into chondrocytes [1], [5] which facilitate deposition of extracellular matrix components at the fracture site resulting in the formation of the transient soft callus [4]. In an initial remodeling phase, the avascular cartilagenous callus is converted into a vascularized and mineralized tissue that is remodeled by osteoclasts during an initial cartilage resorption phase [6], and then later in a bone remodeling phase that sculpts the healed skeletal element into the anatomically appropriate shape [7]–[9]. The importance of vascular invasion during endochondral bone formation has been established, with defects in bone vasculature having been reported in osteoporosis and rickets [10]. Thus, not surprisingly, during skeletal repair, neoangiogenesis driven by vascular endothelial growth factor (VEGF) is required to support nutrient and oxygen transport, with tissue oxygenation being required for osteogenic differentiation [11]–[16]. Further suggesting the need for this angiogenic cascade of events in the repair process, pharmacologic inhibition of angiogenesis has been shown to impair fracture healing by reducing/delaying callus mineralization [17].

G protein coupled receptor kinase 2 (GRK2) interacting protein 1 (GIT1) was originally identified by its binding to GRK2 and its effects on adrenergic receptor endocytosis [18]. GIT1 has five functional domains, including a zinc finger domain responsible for ARF-GAP activity, three ankyrin repeats, a Spa2 homology domain (SHD), a synaptic localization domain (SLD), and a conserved carboxyl-terminal region that interacts with paxillin (PBS) [19]. Through these domains, GIT1 interacts with diverse proteins including ARF6, MEK, phospholipase C-γ (PLCγ), p21-activated kinase (PAK)-interacting exchange factor (PIX) and paxillin [20], [21]. GIT1 has diverse biological functions, which we have shown to include a critical role in pulmonary vascular development by regulating VEGF induced PLCγ and ERK1/2 activation [22]. GIT1 is also upregulated in atherosclerotic plaques and regulates endothelial cell and vascular smooth muscle cell migration [23]. Recently, we identified an important role of GIT1 in bone physiology based on its regulation of osteoclast sealing zone formation, a critical step required for the function of this cell [24]. Based on the observations that GIT1 plays an important role in angiogenesis and osteoclast function, both of which are critical for bone repair, we hypothesized that GIT1 is an important molecular player in the bone healing process.

In the present study, we established that loss of GIT1 alters the fracture healing process. Homozygous GIT1 knockout (GIT1 KO) mice display a persistence of cartilagenous callus evidenced by preservation of Alcian Blue staining and type 2 collagen content. Chondrocyte differentiation was impacted by loss of GIT1, with PCNA and TUNEL staining revealing decreased proliferation and apoptosis of chondrocytes. We used microcomputed tomography (microCT) to investigate overall callus volume and vascular parameters and discovered that GIT1 KO mice have reduced vessel volume and vessel number, which correlated with decreased expression of VEGF-A and VEGFR2. We also examined osteoclst numbers in the callus, and document a reduced number of TRAP-stained cells during the remodeling phases of healing in mice with loss of GIT1 function. Overall, findings documented in this report establish that GIT1 is important for the normal progression of the bone healing program via effects on callus vascularization and osteoclast number, improtant determinants for callus mineralization and remodeling respectively.

## Materials and Methods

### Ethics Statement

To ensure the humane treatment of mice in this study, all experiments involving mice were performed with the approval and supervision of the University Committee on Animal Resources, the AALAC-, OLAW- and USDA-approved IACUC at the University of Rochester Medical Center.

### Animals

Homozygous GIT1 knockout (GIT1 KO) mice were generated on the C57/BL6 background as described in Pang et al [22]. Chimeric mice generated were backcrossed for more than 7 generations. GIT1 KO mice at age of 10–12 weeks were used for femoral fracture with WT littermates used as controls. It is important to note that because of the high rate of perinatal lethality in GIT1 KO mice due to a pulmonary defect [22] and because of sensitivity to anesthesia, we were limited to an experimental strategy that included only 3 KO mice at each of 3 harvest time points post-fracture: 7, 14 and 21 days.

### Mouse Femur Fracture Model

Femur fractures in mice were performed as described in Xie et al [25]. Briefly, mice were anesthetized using a mixture of ketamine and xylazine delivered via intraperitoneal injection. The skin and the underlying tissues over the left knee were incised. A 25-gauge needle was inserted through the patellar tendon and into the medullary canal of the femur. A mid-diaphyseal fracture was created via three-point bending using an Einhorn device [1]. After fracture, 0.5 mg/kg buprenorphine (Abbott labs, North Chicago, Illinois) was administered subcutaneously to each mouse daily for 3 days to control pain. Radiographs were obtained on 7, 14, and 21 days under anesthesia using a Faxitron X-ray system (Faxitron X-ray, Wheeling, Illinois).

### Quantitative Real Time PCR (qPCR)

Fracture calluses from WT mice (n = 4) per time point (7, 14, 21 days) were carefully excised from the lower limb. The soft tissue surrounding the calluses was removed and the samples were flash-frozen in liquid N_2_. Frozen samples were placed in a Tissuelyser (Qiagen, Venlo, Netherlands) along with 1 mL of TRIzol (Invitrogen, Carlesbad, CA). Homogenization was performed using a frequency of 30 Hz for a time of 3 minutes. The samples were checked for adequate disruption and the mRNA was purified according to the TRIzol System protocol. The concentration of stock mRNA was determined in triplicate using a Nanodrop photospectrometer. The mRNA was diluted in RNase-free water and aliquoted into working dilutions of 0.5 µg/µL. A cDNA library was synthesized using 0.5 µg of callus mRNA by a commercially available reverse transcription kit (Invitrogen, Carlesbad, CA). qPCR analyses were performed using murine-specific primers for *GIT1* and *GAPDH*. The qPCR reactions were performed using SyberGreen (ABgene, Rochester, NY) in a RotorGene real time PCR machine (Corbett Research, Carlsbad, CA). GIT1 expression was normalized using GAPDH expression as an internal control.

### Microcomputed Tomography Imaging

Microcomputed tomography imaging (microCT) was performed to assess mineralized callus volume and vascularity as we have previously described [25]–[28]. Vascular networks at the cortical bone junction and around the fractures were examined using microCT analysis combined with perfusion of a lead chromate based contrast agent [29]. Briefly, Microfil MV-122 (Flow Tech, Inc., Carver, Massachusetts) contrast media, a radiopaque silicone rubber compound containing lead chromate, was perfused via the heart along with 4% paraformaldehyde following an initial vascular flush with heparinized saline. After perfusion, the fractured femur was removed and scanned using a microCT imaging system (VivaCT 40; Scanco Medical AG, Basserdorf, Switzerland) at resolution of 10.5 µm to image bone and vasculature. The samples were subsequently decalcified for 21 days using a 10% EDTA solution. After complete decalcification, the samples were scanned again to image only vascularization within the callus. By registering the 2-D slices before and after decalcification, contour lines were drawn to define a VOI that only included the vasculature in or immediately adjacent to the fracture callus itself. The reconstructed scan images were globally thresholded based on intensity values to render 3-D images of the vasculature in new bone callus, excluding the vessels in the surrounding tissues. Three-dimensional morphometric analysis, based on direct distance transform methods, was subsequently performed on the 3-D images using algorithms that are commonly used to model trabecular bone morphology. This facilitated quantification and analysis of vascular network morphology including vessel volume, number, spacing, and connection density. It should be noted that all measurements of the vascular network were constrained by the limit of resolution of the scanner (10 µm) and the permeability of the networks to the viscous Microfil. Careful heparinization and consistent application of perfusion conditions were established to ensure comparability between mice.

### Histology and Histomorphometry

Fractured femora harvested for microCT analysis were processed for histology as previously described [30]. Briefly, femora were disarticulated at the knee and hip, denuded of soft tissue, and fixed at RT in 10% NBF for 72 hours. After three washes in phosphate buffered saline (PBS), fixed femora were decalcified in 10% EDTA for 7 to 14 days. Tissues were then processed using a Tissue-Tek VIP 6 tissue processor (Sakura Finetek USA, Inc., Torrance, CA, USA) and embedded in paraffin. Serial 3 µm thick sagittal sections were obtained from a 60 µm region spanning the center of the fracture callus. Three sections, each separated by approximately 25 to 50 µm, were stained with Alcian Blue Hematoxylin/Orange G and histomorphometric analysis was performed using a point counting method as described previously [31]. Briefly, blinded sections were analyzed using a standardized eyepiece grid under the 10× objective to determine the percent of total callus area composed of cartilage and woven bone. Cartilage was defined as tissue with positive Alcian Blue stain. Woven bone was counted whenever a trabecular structure was observed, regardless of staining. Cortical bone and internal (i.e. intramedullary) callus were not included in these analyses. At each intersection of a horizontal and vertical grid line, the identity of the underlying tissue was determined, with the outcomes for every intersection documented and counted. Based on the number of counted intersections on each slide, the relative area of each tissue type as a percentage of the callus area (i.e. grid intersections that fell within the callus domain) was calculated.

### Immunohistochemistry

Previosly published methods were employed for immunohistochemical analysis of PCNA and VEGFR2 expression [32] and type 2 collagen expression [33]. Briefly, for either antigen, sections were incubated at 60°C for 30 minutes, followed by deparaffinization in xylene and hydration in gradient ethanol. Sections were permeabilized in PBS containing 0.2% Triton X-100 (Sigma, St. Louis, MO) for 10 min, washed in PBS, blocked with 3% H_2_O_2_ in methanol for 30 minutes, and 3% goat serum in PBS for 30 minutes. Sections were then incubated with primary antibodies overnight at 4°C. The next day, sections were washed with PBS and incubated with biotinylated secondary antibodies (Vector Laboratories, Burlingame, CA) in blocking buffer for 30 minutes. After washing with PBS, ABC reagent (Vector Laboratories) was added for 30 minutes and sections were washed again before detection with AEC reagent (Vector Laboratories). Negative controls were performed on adjacent sections by omitting primary antibodies. All sections were analyzed using an Olympus VS120 Whole Slide Imager and quantification was performed using the automated Visiopharm Integrator System (Visiopharm, Hoersholm, Denmark) and its associated software.

### Immunofluorescence

Slides from WT and GIT1 KO femure fractures were deparaffinized in xylene, rehydrated in graded ethanol, and rinsed in distilled, deionized H_2_O as described above. For PECAM1 (CD31) detection, slides were boiled for 15 minutes in citrate buffer (Zymed Laboratories, San Francisco, CA), cooled for 20 minutes, and washed in PBS for 5 minutes. Slides were then blocked with normal goat serum containing 5% BSA in PBS for 30–60 minutes and incubated overnight with primary antibodies diluted 1∶100 in blocking buffer. After two rinses with PBS, the sections were incubated in the dark for 1 hour at 37°C with rabbit secondary antibody diluted 1∶500 in normal rabbit serum. Slides were rinsed in PBS before the addition of Topro3 nuclear stain (Invitrogen, Grand Island, NY) and then mounted with Prolong Gold mounting media (Invitrogen). Confocal images were captured using Zeiss LSM 510 Axioskop 2 microscope (Zeiss Microimaging, Thornwood, NY) and analyzed with Zen 2007 software (Zeiss, San Diego, CA).

### Osteoclast Quantification

Three sections per callus were stained for tartrate-resistant acid phosphatase (TRAP) using a previously described method [34]. Briefly, after deparaffinization and rehydration with distilled water, sections were incubated at 37°C for 25 minutes in a solution of anhydrous sodium acetate (Sigma), L-(+) tartaric acid (Sigma), glacial acetic acid, fast red violet LB salt (Sigma), naphthol AS-MX phosphate (Sigma), ethylene glycol monoethyl ether (Sigma), and distilled water. Sections were rinsed in distilled water, counterstained with hematoxylin for 10 seconds and then placed in ammonia water for 5 seconds. Quantification was completed using the 10× objective and Osteomeasure software (OsteoMetrics, Inc., Decatur, GA) to contour bone perimeter within the callus and identify osteoclast number as a percentage of covered bone surface perimeter and as a number of cells per mm of bone surface. Osteoclasts were defined as multi-nucleated, TRAP-positive cells seated on a bone surface.

### Statistical Analysis

All values are expressed as mean +/− SEM. Statistical differences between groups were detected using either ANOVA (when >2 experimental groups were compared) or two-tailed unpaired Student’s t tests (when only 2 experimental groups were compared). A p-value less than 0.05 (p<0.05) was considered significant.

## Results

### GIT1 is Expressed during Fracture Healing

**Figure 1:**
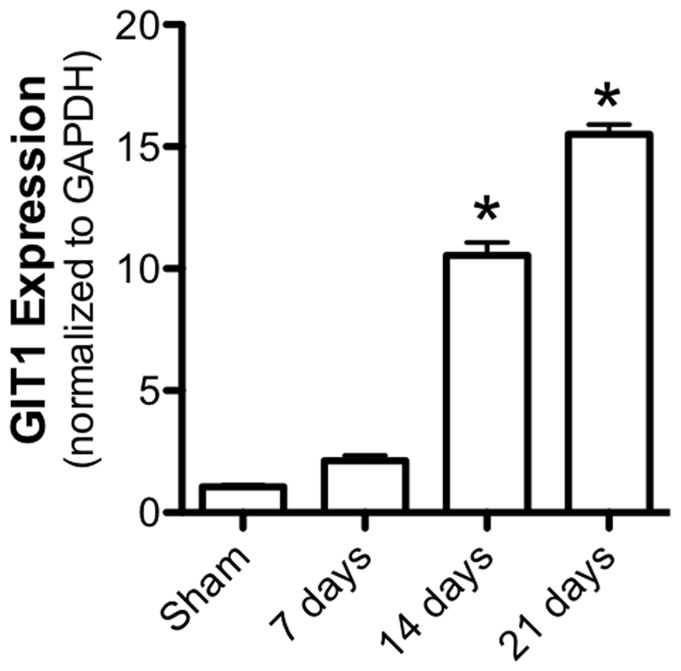
GIT1 mRNA is expressed during fracture healing. WT mice were administered femur fractures and fractured femora were harvested for isolation of mRNA from the callus at 7, 14, and 21 days post-injury. qPCR was performed to examine the profile of GIT1 expression. Bars represent mean GIT1 expression level relative to GAPDH +/− SEM (N = 4, *p<0.05).

To establish that GIT1 is expressed and regulated during the fracture healing process, qPCR was performed on mRNA isolated from the healing callus of WT mice at days 7, 14 and 21 post-fracture. Consistent with previously published work identifying GIT1 function in vascular tissue and osteoclasts, GIT1 is upregulated significantly by day 14 and remains highly-expressed at day 21 (Fig. 1). These timepoints correspond to callus revascularization, cartilage remodeling and woven bone remodeling in the temporal progression of healing. These results set the stage for study of bone healing in mice in the context of GIT1 loss-of-function.

### Fracture Healing is Impaired in GIT1 KO Mice

**Figure 2:**
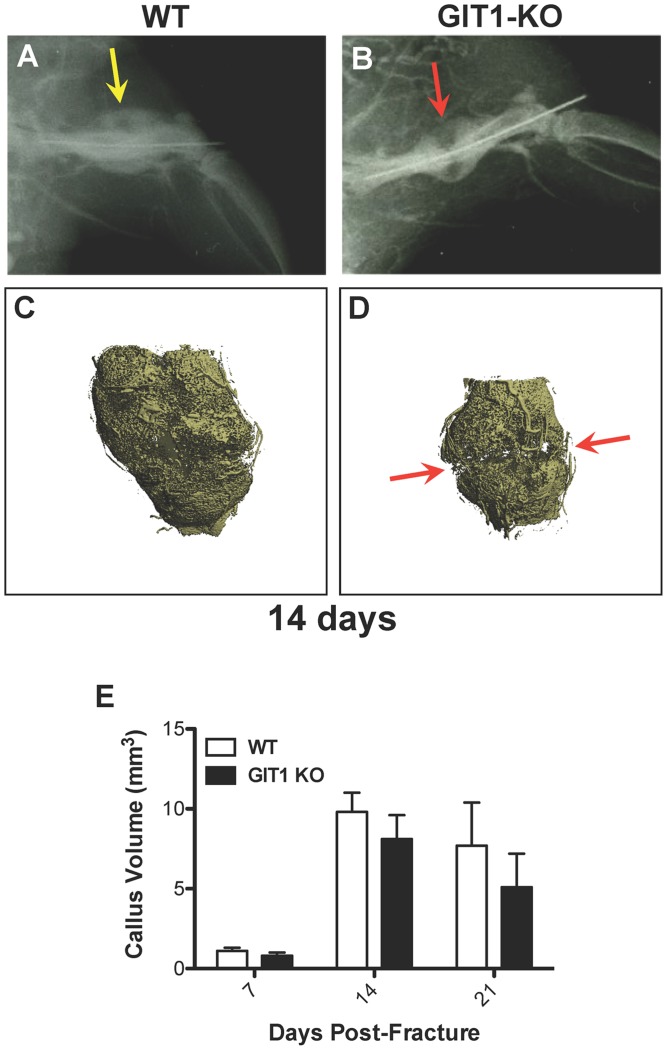
Disjunction persists at 14 days post-fracture in GIT1 KO mice. Femur fractures were induced in 10-week-old WT and GIT1 KO mice. Fractured femora were harvested for analysis at 7, 14, and 21 days post-injury. Radiographs obtained at the 14 day time point consistently revealed radiolucency in GIT1 KO calluses (B, red arrow) compared with calluses from WT mice (A, yellow arrow). This was supported by microCT analysis, which revealed lack of bridging mineral in GIT1 KOs (D, red arrows) compared to a connected shell of mineral in WT controls (C). Further quantification of callus geometry via microCT indicated that there were no differences in mineralized callus volume between WT and GIT1 KO mice (E). Bars represent mean callus volume (mm^3^) +/− SEM (N = 3, *p<0.05).

To begin investigating the role of GIT1 in the fracture healing process, we induced femoral fractures in WT and GIT1 KO mice and assessed the healing process by radiographical evaluation and microCT analysis of mineralized callus volume. At day 14, loss of radiolucency at the fracture site in WT mice suggested the normal pacing of repair (Fig. 2A), while the healing process was delayed in GIT1 KO mice (Fig. 2B, red arrow). Representative microCT reconstructions confirm persistence of disjunction in GIT1 KO mice (Fig. 2D, red arrows indicating disjunction) compared to WT mice (Fig. 2C) at 14 days post-fracture, although quantification of mineralized callus volume in cohorts of animals failed to achieve significance at any timepoint, only revealing a trend toward a decrease (Fig. 2E).

### Chondrocyte Maturation is Delayed in Fracture Callus of GIT1 KO Mice

**Figure 3:**
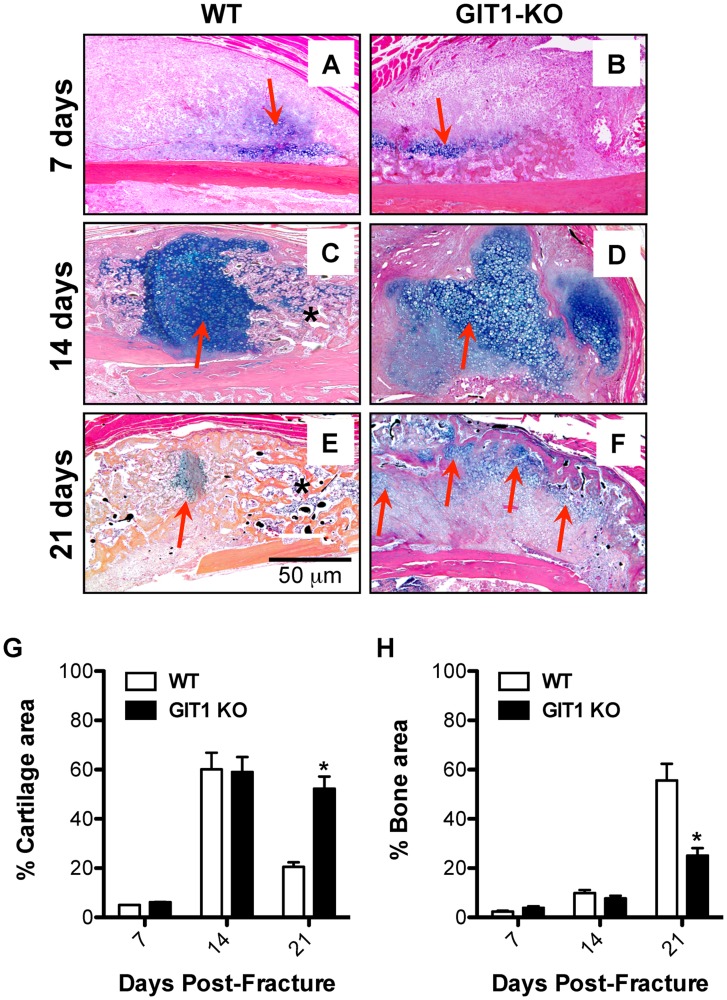
Cartilage persists and woven bone callus is delayed in GIT1 KO mice. Histological analysis of fracture callus cartilage content was performed via Alcian Blue Hematoxylin/Orange G staining. Representative stains of calluses from WT and GIT1 KO mice at 1, 2 and 3 weeks post-fracture are displayed (A–F). Red arrows denote Alcian Blue-stained cartilagenous matrix and asterisks denote mineralized woven bone. Histomorphometry was performed on triplicate sections from multiple mice, with % Cartilage Area (G) and % Bone Area (H) quantified. Bars represent % Area (cartilage or bone) +/− SEM (*p<0.05, N = 3).

Alcian Blue was used to stain extracellular matrix surrounding chondrocytes within the fracture callus, with representative sections depicted (Fig. 3A–F). At days 7 and 14 post-fracture, cartilage matrix content in WT mice (Fig. 3A and 3C) was similar to that in GIT1 KO mice (Fig. 3B and 3D). However, unlike in WT mice (Fig. 3E), cartilaginous callus in GIT1 KO mice was still present at day 21 (red arrows, Fig. 3F). Histomorphometric quantification of callus cartilage and bone content confirmed that at 21 days post-fracture, GIT1 KO mice had persistent cartilage (Fig. 3G) that was at the expense of woven bone (Fig. 3H).

**Figure 4:**
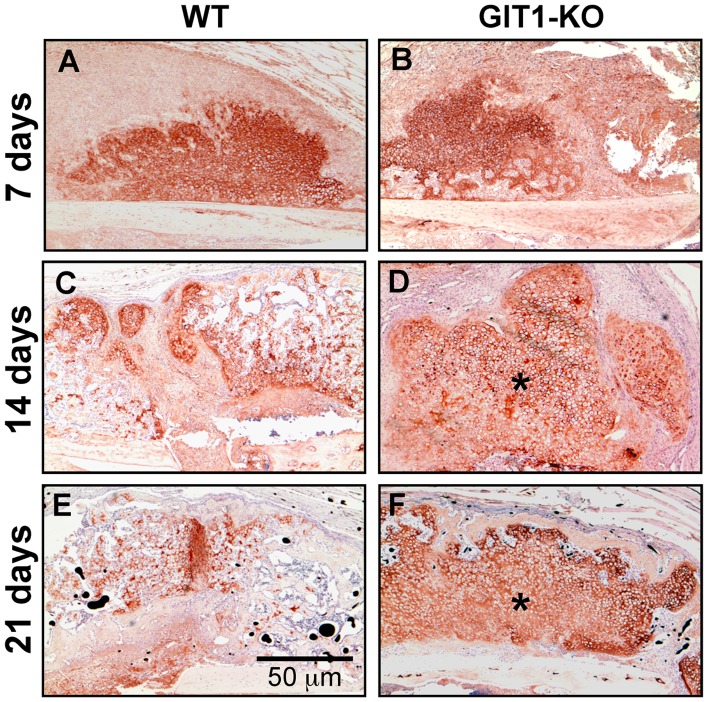
Type 2 collagen-containing matrix persists in GIT1 KO mice. Tissue sections cut from WT and GIT1 KO mice were analyzed for COL2A1 content using an immunohistochemistry approach. Representative stains at 7, 14 and 21 days post-fracture are depicted, with asterisks denoting areas within the callus at 2 and 3 weeks post-fracture in GIT1 KO mice (D and F respectively) that have more robust/persistent staining.

To further examine the cartilage phenotype, we performed COL2A1 immunohistochemistry in WT and GIT1 KO mice at 7, 14 and 21 days post-fracture. Representative stained sections are displayed in Fig. 4, with GIT1 KO mice trending toward persistence of COL2A1 at 14 and 21 days (Fig. 4D and 4F) compared to matched sections from WT mice (Fig. 4C and 4E). These results suggest that cartilage persists in the fracture callus of GIT1 KO mice at the expense of woven bone formation.

### Chondrocyte Proliferation and Apoptosis is Reduced in Fracture Callus of GIT1 KO Mice

**Figure 5:**
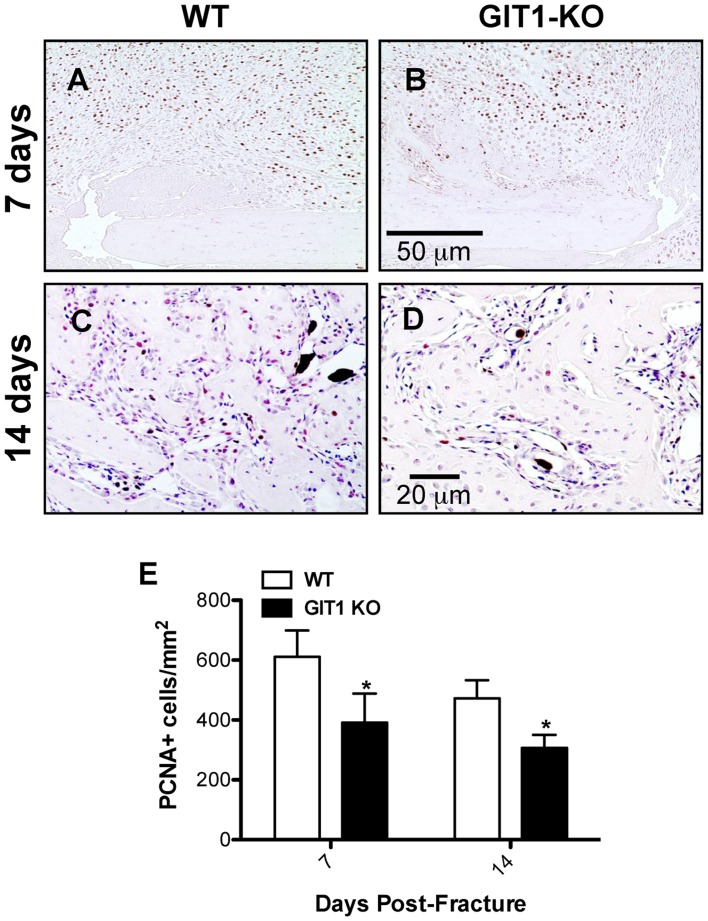
Chondrocyte proliferation is reduced in GIT1 KO mice. Representative PCNA staining is shown at 7 and 14 days post-fracture in WT mice (A and C) and at 7 (B) and 14 days (D) post-fracture in GIT1 KO mice (B and D respectively). Histomorphometry was performed on triplicate sections from multiple mice to quantify the number of PCNA-positive cells per unit callus area (E). The data is presented as mean of the number of PCNA positive cells/mm^2^+/− SEM (*p<0.05, N = 3).

**Figure 6:**
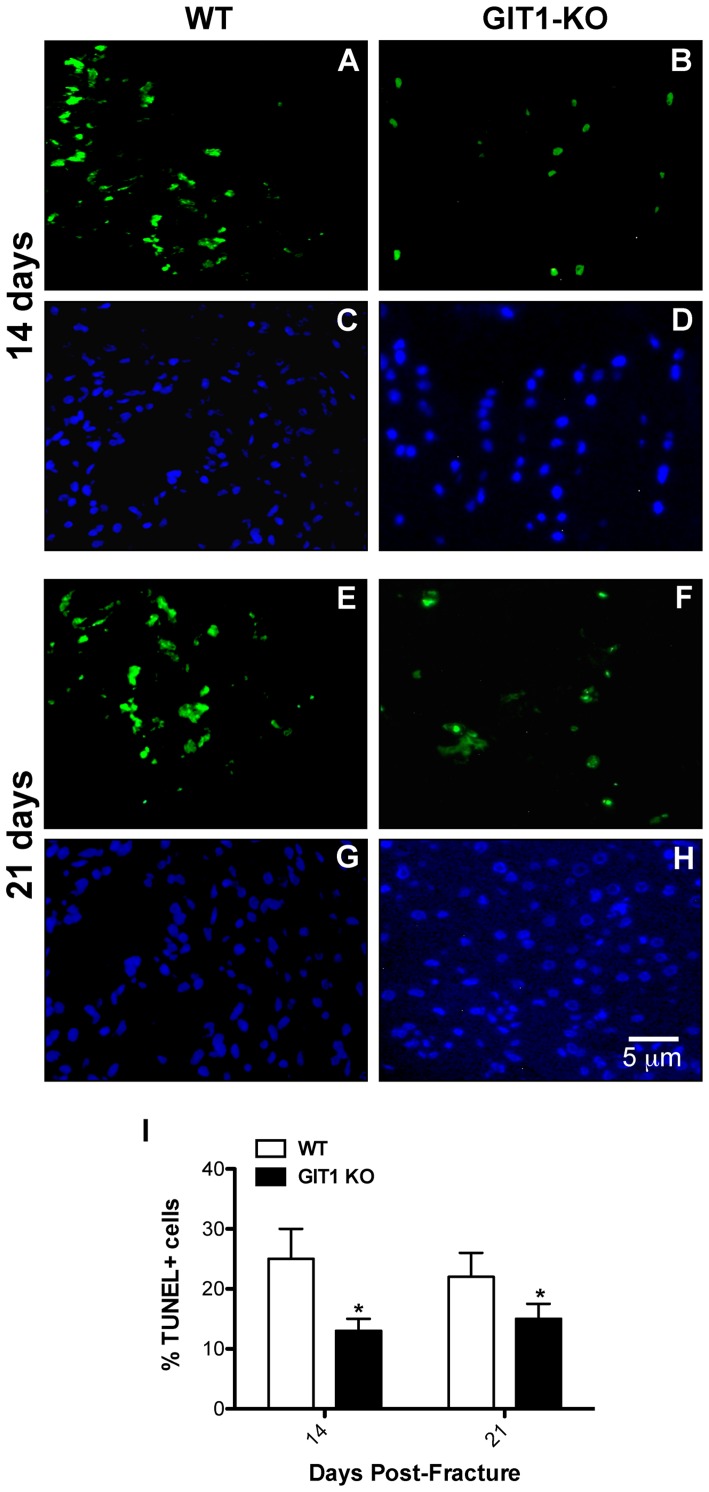
Chondrocyte TUNEL staining is reduced in GIT1 KO mice. Chondrocyte apoptosis was assessed in WT and GIT1 KO mice at 14 and 21 days post-fracture. Representative TUNEL immunofluorescence and DAPI staining at both time points is presented in WT mice (A/C and E/G respectively) and in GIT1 KO mice (B/D and F/H respectively). Quantitative histomorphometric analyses of the number of TUNEL-positive cells per unit area in triplicate sections from three WT and GIT1 KO mice at 14 and 21 days post-fracture are presented (I). Bars represent the percent of TUNEL positive cells/mm^2^+/− SEM (*p<0.05, N = 3).

To explore the possible association between persistent cartilagenous callus and changes in chondrocyte proliferation and apoptosis, we performed PCNA immunostaining and TUNEL immunofluorescence respectively. At days 7 and 14, PCNA-positive cells were more abundant in the cartilaginous soft callus of WT mice (Fig. 5A and 5C) relative to calluses from GIT1 KO mice (Fig. 5B and 5D). Histomorphometry confirmed this, establishing that GIT1 KO chondrocytes were less proliferative at 7 and 14 days post fracture (Fig. 5E). Regarding apoptosis, representative TUNEL-stained sections reveal that the percentage of TUNEL-positive cells (relative to DAPI) was reduced in GIT1 KO chondrocytes at both day 14 and 21 post-fracture (Fig. 6A–H). Again, this was confirmed quantitatively (Fig. 6I), indicating that GIT1 KO chondrocytes are less apoptotic during the time period of healing that normally involves remodeling of the cartilagenous callus (i.e. conversion to bone) and associated programmed death of chondrocytes. While GIT1 appears to have a role in modulating proliferation and death of chondrocytes, it is not clear how these combined phenotypes (reduced mitosis and death in the GIT1 KO group) account for the net persistence of the cartilagenous callus and the delay in its conversion to woven bone.

### Blood Vessel Volume and Number are Decreased in Fracture Callus of GIT1 KO Mice

**Figure 7:**
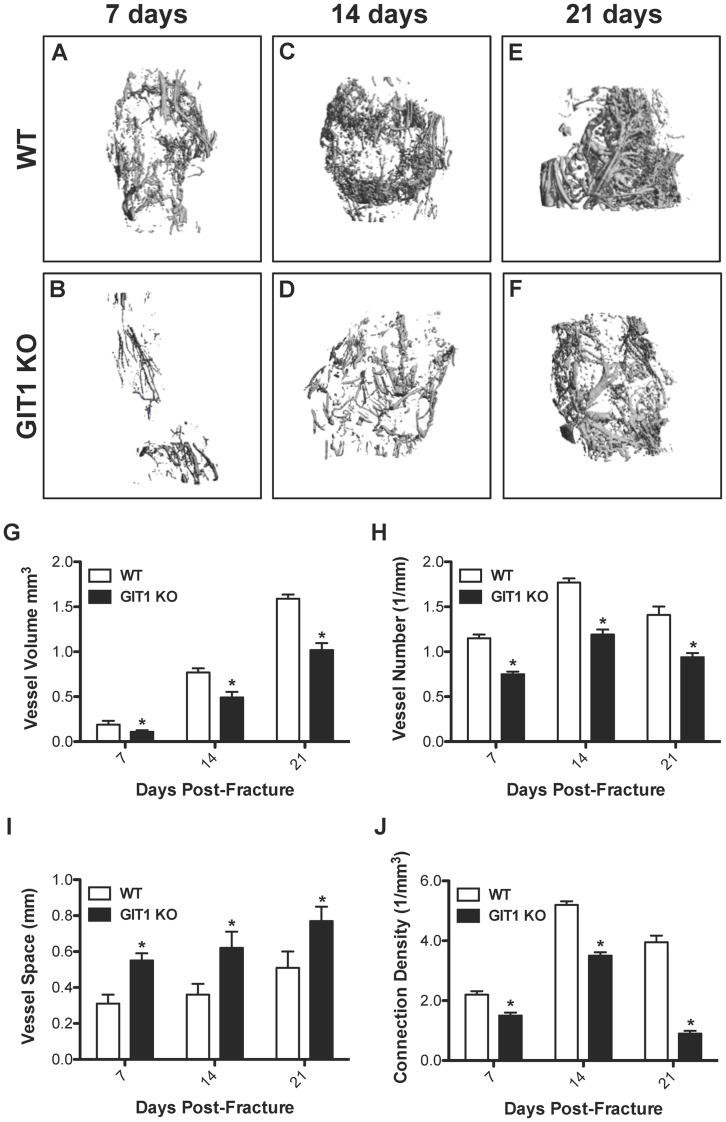
Fracture callus vascularity is reduced in GIT1 KO mice. To visualize and quantify callus vascularity, WT and GIT1 KO mice were perfused with lead chromate microfilm perfusion reagent. Harvested femora were decalcified and representative vascular microCT reconstructions from each experimental group at 7, 14 and 21 days post-fracture are presented. Reduced vascularity in GIT1 KO mice (A, C, E) compared to WT control mice (B, D, F) was evident at all time points. Quantification of callus vascular parameters, including Vessel Volume (G), Vessel Number (H), Vessel Spacing (I) and Connection Density (J) supported these findings, with GIT1 KO mice possessing reduced callus vessel volume, vessel number and connection density and increased space between vessels compared to callus from WT mice. Bars represent mean for each value +/− SEM (N = 3, *p<0.05).

Based on our previously published results demonstrating a pulmonary vascular deficit in GIT1 KO mice, we hypothesized that delayed bone repair in these animals could be due to impaired angiogenesis in the fracture callus. Therefore, we performed quantitative vascular microCT analyses to evaluate neovascularization at day 7, 14, and 21 post-fracture in WT and GIT1 KO mice. Representative reconstructions indicated reduced callus vascularity in KO mice compared to the WT cohort, with GIT1 KO mice displaying a marked reduction in vessel volume, number, and connectivity (Fig. 7A–F). These apparent changes were supported by quantifation of various vascular parameters, substantiating that compared to the WT cohort, GIT KO mice had reduced vessel volume (Fig. 7G), reduced vessel number (Fig. 7H), increased spacing between vessels (Fig. 7I) and reduced connection density (Fig. 7J). It should be noted that the quantification of reduced vessel connectivity (i.e. connection density) is influenced by the efficiency of vessel perfusion with contrast agent, and there may not be adequate filling in vascular beds with smaller vessel diameters, reducing the accuracy of the connectivity algorithm and conclusions that are drawn from it.

### Neovascularization is Impaired in the Fracture Callus of GIT1 KO Mice

**Figure 8:**
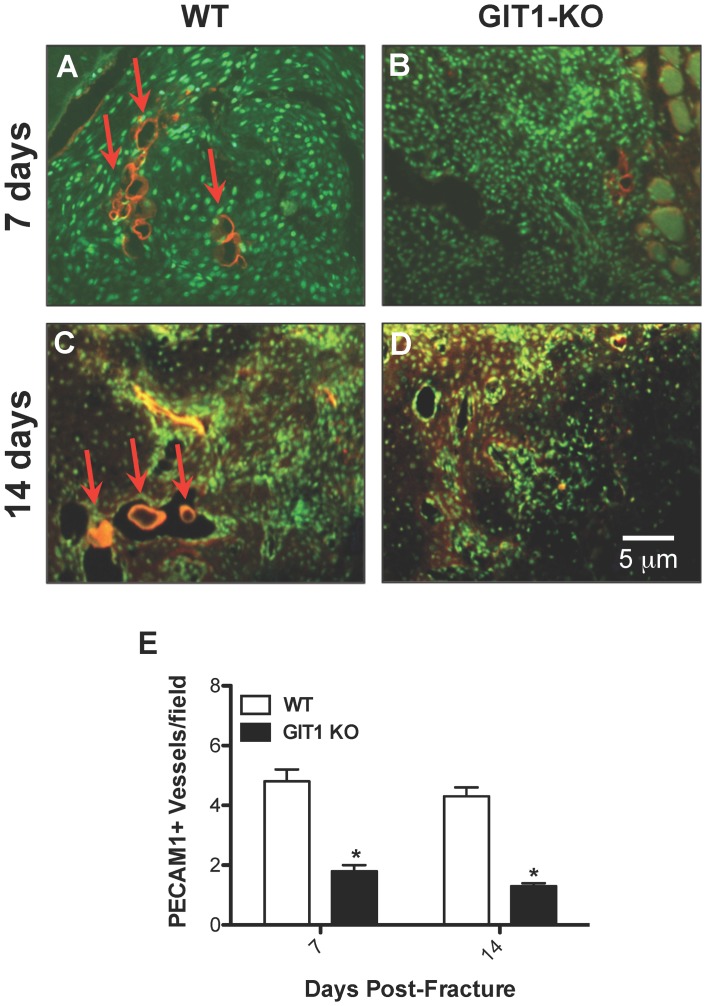
PECAM1^+^ blood vessel number is reduced in GIT1 KO mice. Representative PECAM1 immunofluorescence is presented at 7 and 14 days post-fracture in WT mice (A and C) and GIT1^−/−^ mice (B and D). Histomorphometry was performed to quantify the average number of positively-stained blood vessels present in each field of view on each section analyzed. Three sections (from 3 levels within each callus, 25–50 µm apart) were viewed using the 10× objective, with counts being collected from 3 fields of view in each section. All counts from each callus (9 fields total) were averaged. Vessel counting using this approach confirmed the immunofluorescence in panels A–D, with WT calluses possessing between 2 and 3-fold more PECAM1^+^ vessels than GIT1 KO calluses at both time points (E). Bars represent mean number of PECAM1^+^ vessels/field +/− SEM (*p<0.01, N = 3).

**Figure 9:**
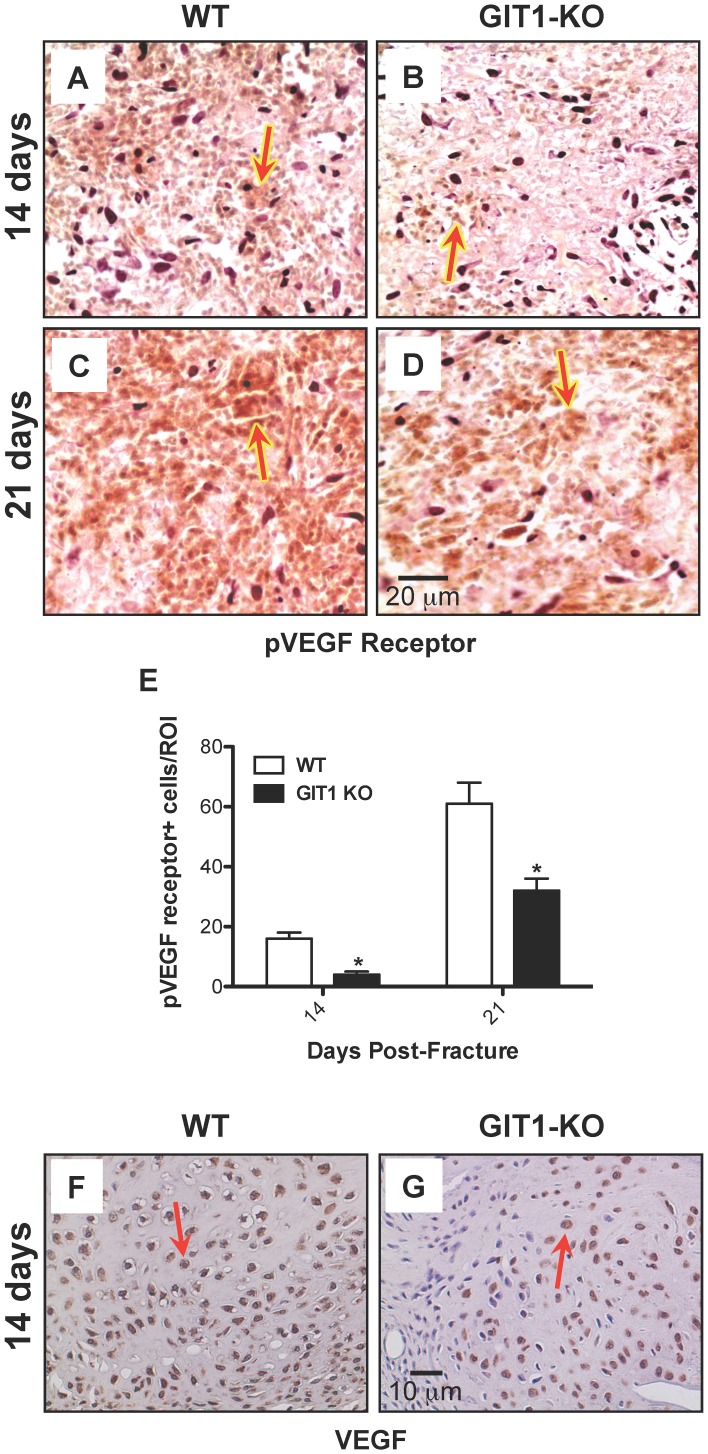
VEGF signaling is reduced in GIT1 KO mice. Phospho-VEGF receptor immunostaining was performed on fracture calluses from WT and GIT1 KO mice at 2 and 3 weeks post-fracture. Panels A-D depict representative staining profiles, with Phospho-VEGF receptor-positive cells staining red as indicated by red arrows. Histomorphometry was performed to quantify the number of Phospho-VEGF receptor-positive cells per unit callus area (E). Data is presented as the mean number of positive cells per unit area (i.e. region of interest) +/− SEM (*p<0.01, N = 3). Additionally, immunohistochemistry was performed of assess VEGF levels in fracture calluses from WT (F) and GIT1 KO mice (G). Representative histological sections of calluses at 2 weeks post-fracture are presented, depicting reduced expression in KO mice. VEGF positive cells are stained reddish-brown as indicated by red arrows.

To further evaluate angiogenesis, we analyzed vessel number using an antibody against PECAM1 (CD31), an endothelial cell marker. A strong signal was observed in vessels of the callus from WT mice at days 7 and 14 (Fig. 8A and 8C). In contrast, the number of PECAM1^+^ vessels was dramatically reduced in GIT1 KO mice (Fig. 8B and 8D). Quantification of the number of blood vessels revealed a 60% reduction in GIT1 KO mice (Fig. 8E) at both 7 and 14 days post-fracture. In order to examine why vessel parameters (from the vascular microCT findings, Fig. 7) and vessel density (PECAM1) were lower in GIT1 KO mice, we first analyzed the amount of phospho-VEGFR2 expressed in the callus. Compared to WT mice, the phospho-VEGFR2 positivity was reduced in GIT1 KO mice at 14 and 21 days post-fracture (Fig. 9A–D). Quantification in multiple mice from each cohort supported this, revealing a 50% reduction in GIT1 KO mice at days 14 and 21 (Fig. 9E). Since the active form of VEGF receptor was significantly decreased at 14 and 21 days, we next examined VEGF expression levels. Representative immunohistochemistry at 14 days post-fracture depicts reduced VEGF expression in GIT1 KO callus compared to WT controls (Fig. 9F–G). Taken together, these findings indicate that GIT1 is critical for VEGF induced microvessel sprouting in fracture callus, and GIT1 deficiency impairs neoangiogenesis in response to injury.

### Osteoclast Number is Reduced in GIT1 KO Fracture Callus

**Figure 10:**
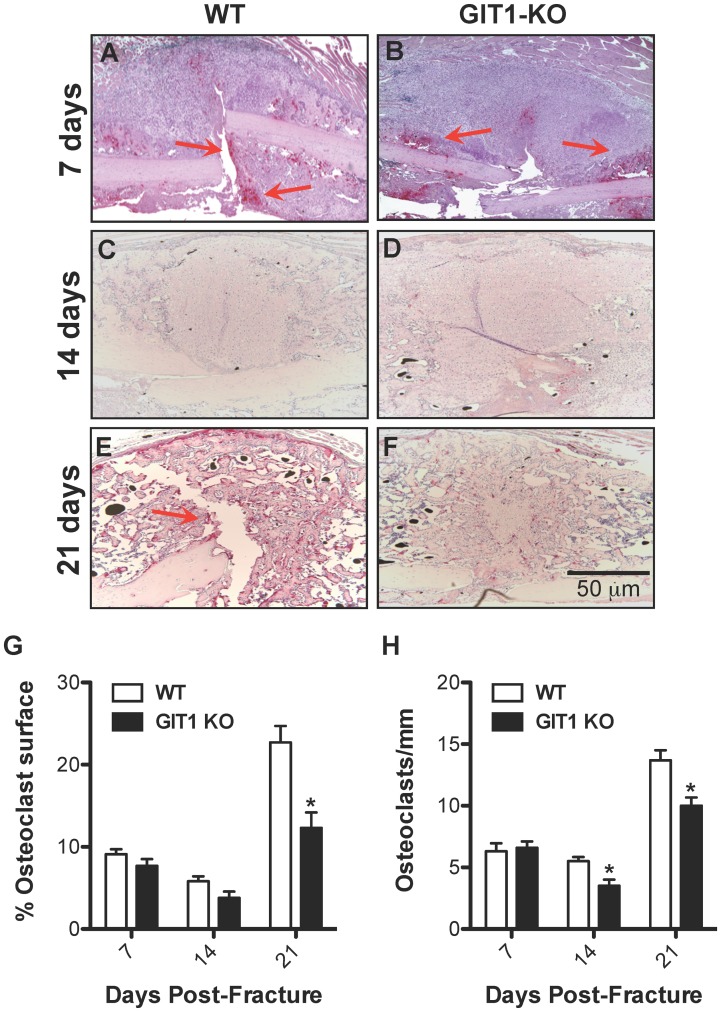
Osteoclast number is reduced in GIT1 KO mice. The presence of osteoclasts in the fracture callus of WT and GIT1 KO mice was assessed at 7 (A and B respectively), 14 (C and D respectively) and 21 days (E and F respectively) post-fracture via TRAP staining. Histomorphometry to quantify percentage of osteoclast surface (G) and osteoclast number per unit bone surface (H) was also performed on triplicate sections from multiple mice, with bars representing the mean for each parameter +/− SEM (*p<0.05, N = 3).

As mentioned, alterations in callus chondrocyte proliferation and apoptosis cannot explain the healing phenotype seen in GIT1 KO mice. Since we have previously established that osteoclast differentiation and activity is impaired in GIT1 KO mice [24], we therefore focused on assessing osteoclast numbers. To assess osteoclast number, histology-based quantification of the number of TRAP-positive cells was performed in callus samples from both WT and GIT1 KO mice at days 7, 14 and 21 post-fracture. Although there was no difference in the number of the TRAP positive cells and the amount of cartilaginous callus (Alcian blue) between WT (Fig. 10A and 10C) and GIT1 KO mice (Fig. 10B and 10D) mice at day 7, differences were observed at days 14 and 21. The increased percentage of cartilaginous callus in GIT1 KO mice compared to WT mice at day 21 (Fig. 3) was concordant with what appeared to be a marginally decreased TRAP positivity in the GIT1 KO cohort at day 14 (Fig. 10C, 10D, 10G and 10H), and a significant decrease in TRAP staining at day 21 (Fig. 10E, 10F, 10G and 10H). This net reduction in osteoclast number in GIT1 KO mice could account for the larger amount/persistence of cartilage callus in these animals that we have observed via Alcian Blue staining (seen in both Fig. 10 and Fig. 3).

## Discussion

Delayed and/or failed fracture repair are major public health issues that impact populations in the United States and throughout the world. Musculoskeletal deficits that result from delayed healing post-injury and post-surgery limits the performance of normal daily activities, compromises the function of various organ systems, and has a debilitating impact on psychological health and social function [35]–[41]. Given the scope of this public health crisis, it is the goal of researchers in the field to further understand the molecular basis for normal and pathologic healing with the aim of developing novel therapeutic strategies.

Endochondral-based healing, which predominates in the repair of long bones, requires the correct temporospatial coordination of a series of molecular and cellular events [42]. From a tissue architecture perspective, these events are organized into four overlapping phases – (1) inflammation, (2) soft (cartilaginous) callus formation, (3) resorption of cartilage and hard (woven bone) callus formation, and (4) woven bone remodeling. Mesenchymal stem cells (MSCs) and chondro−/osteo-progenitor populations that primarily reside in the periosteum are essential throughout fracture repair [43], [44]. The transition from soft to hard callus requires angiogenesis, because increased tissue oxygen concentration is necessary for osteoprogenitors to mineralize the matrix. Additionally, functional osteoclasts are needed to remove the cartilage matrix during woven bone formation and to remodel the woven bone callus to ultimately recapitulate the normal structure of the injured element.

Our group has recently documented a critical role for GIT1 in pulmonary vascular development [22] and in the formation of functional osteoclasts via regulation of sealing zone formation [24]. Given these unique roles for GIT1, both critical for normal tissue morphogenesis in fracture repair, we became interested in the function of this protein during the bone healing process. Based on our previous work, we hypothesized that loss of GIT1 function will lead to delayed healing in a mouse model of femur fracture due to i) impaired neovascularization of the callus and ii) inhibited cartilage and woven bone remodeling due to reduced osteoclast number and/or function. Supporting this hypothesis, our results indicate that GIT1 deficiency leads to a fracture healing defect that is driven by two primary effects: altered neovascularization of the callus in early-mid stage healing, and reduced osteoclast number during primary and secondary callus remodeling.

Regarding blood vessels in the callus, successful bone repair requires the revascularization of injured tissues to provide oxygen, facilitate metabolic waste management, and deliver a population of circulating precursor cells that may contribute to healing either directly or in a paracrine manner. Angiogenesis during the repair process is thought to be modulated by VEGFs and their cognate receptors VEGFR1 and VEGFR2. It has been demonstrated that delivery of VEGF during mouse femur fracture healing enhances vascular ingrowth into the callus and accelerates repair by promoting bony bridging [45]. This has been confirmed in allograft bone healing, where VEGF gene therapy accelerates the healing process [26]. Conversely, inhibition of new blood vessel formation by injecting TNP-470, an endostatin-like anti-angiogenesis agent [46], prevented fracture healing in a rodent fracture model [17]. Here, we show for the first time that GIT1 may be an important regulator of angiogenesis during fracture repair, thus having a direct impact of the progression of the healing process. Previously we demonstrated that GIT1 is required for activation of PLC-γ and ERK1/2 in endothelial cells and osteoblasts, leading to the regulation of VEGF expression in osteoblasts through the GIT1-ERK1/2 signaling axis [47]. In the present study, we demonstrate that loss of GIT1 results in reduced VEGF expression and phospho-VEGFR2 (active form) in the fracture callus. We also found that GIT1 deficiency significantly decreased small vessel connectivity density and PECAM1 expression in the fracture callus. Correlated with this, vascular microCT analyses revealed reduced overall vessel volume, number and connection density coupled with increased spacing between vessels. These results suggest that the impaired fracture healing process in GIT1 KO mice is at least in part related to impaired VEGF-induced angiogenesis, implicating GIT1 as central regulator of angiogenesis in the context of bone healing.

In addition to the resulting vascular defect described above, the loss of GIT1 function likely also contributes to impaired fracture healing due to altered primary and secondary remodeling because of a defect in osteoclast formation and/or function. We have previously published that GIT1 is required for appropriate osteoclast function via its role in regulating cytoskeletal-related ruffled border and sealing zone formation [24]. Following the formation of the cartilagenous callus, an initial phase of osteoclast-driven remodeling removes the cartilage template, which is then replaced by mineralized woven bone matrix. This occurs in response to macrophage colony-stimulating factor (M-CSF), RANK ligand (RANKL) and osteoprotegerin (OPG) [6]. This initial woven bone matrix is subsequently replaced by organized lamellar bone through a second remodeling process that is the final step in achieving an anatomically correct skeletal element. This second remodeling process is also governed by osteoclasts, which become dominant in this final stage due to the induction of IL-1 and TNF-α and the subsequent expansion of the functional osteoclast population [7], [8] via RANKL in the remodeling callus [9]. In this report, histomorphometric analysis revealed persistent cartilaginous callus that could be the result of delayed cartilage matrix removal due to reduced osteoclast number (and possibly activity) in GIT1 deficient mice that was seen at day 14 and 21 (i.e. during primary and secondary callus remodeling). This phenotype could be related to both the reduced number of osteoclasts observed in the callus area as well as reduced formation of resorbing zones (ruffled border), a known phenotype following loss of GIT1 [24]. Overall, these findings suggest that osteoclast-dependent callus remodeling is likely at least partially impaired in GIT1 KO mice. The molecular basis of this effect may involve several mechanisms including altered RANKL and OPG expression, or cytoskeletal defects that alter osteoclast function, a subject requiring further study.

In addition to these central defects in fracture healing seen in GIT1 KO mice, we also observed an alteration in normal chondrogenic differentiation and cartilage persistence. While expression of Sox9, the master inducer of chondrogenesis, was not altered (data not shown), there was reduced chondrocyte proliferation and apoptosis (Fig. 7 and 8). This was in conjunction with enhanced Alcian Blue staining and cartilage persistence coupled with delayed woven bone formation (Fig. 5) and type 2 collagen immunoreactivity (Fig. 6). Since it is not known if there is a direct role for GIT1 in normal chondrocyte physiology, we postulate that these defects could be indirect and downstream of impaired neovascularization of the callus and/or reduced osteoclast formation and function. Further effort is required to determine any potential direct effects of GIT1 deficiency on chondrocyte differentiation.

In conclusion, data is presented in this report that supports a previously unappreciated role for GIT1 as a regulator of bone fracture healing. GIT1 deficiency leads to decreased revascularization of the fracture callus, decreased chondrocyte proliferation and apoptosis, and reduced osteoclast number. Since the fragility of the GIT1 KO model severely limited the completion of a full evaluation of healing including a higher N for histologic and microCT analyses, mRNA profiling of the callus to further establish molecular mechanism, and performance of biomechanical testing, further study into the role of GIT1 in fracture repair is required. The development of a floxed GIT1 KO model allowing temporal and tissue-specific gene ablation would facilitate the next step in the study of mechanism that would alleviate the high rate of mortality leading to the low number of mice available to populate the experimental groups included in this study. Despite this shortcoming, the results presented clearly define GIT1 as a contributor to bone repair. We speculate that agents targeting activation of GIT1 could be exploited to i) improve callus vascularization in the early phases of healing, and ii) accelerate osteoclast-driven remodeling steps, in particular the resorption of cartilagenous callus that is required to clear the path for deposition of woven bone and stabilization of the fracture site.
